# Association of blood lipids, creatinine, albumin, and CRP with socioeconomic status in Malawi

**DOI:** 10.1186/1478-7954-11-4

**Published:** 2013-02-28

**Authors:** Iliana V Kohler, Beth J Soldo, Philip Anglewicz, Ben Chilima, Hans-Peter Kohler

**Affiliations:** 1 Population Studies Center, University of Pennsylvania, 3718 Locust Walk, Philadelphia, PA 19104, USA; 2 Department of International Health and Development, Tulane University, 1440 Canal St., New Orleans, LA 70112, USA; 3 Community Health Sciences Unit, Ministry of Health and Population, Private Bag 65, Lilongwe, Malawi

**Keywords:** Biomarkers, Blood lipids, Creatinine, Albumin, Wide-range CRP, Socioeconomic status, Variation, Malawi

## Abstract

**Background:**

The objective of these analyses is to document the relationship between biomarker-based indicators of health and socioeconomic status (SES) in a low-income African population where the cumulative effects of exposure to multiple stressors on physiological functions and health in general are expected to be highly detrimental for the well-being of individuals.

**Methods:**

Biomarkers were collected subsequent to the 2008 round of the Malawi Longitudinal Study of Families and Health (MLSFH), a population-based study in rural Malawi, including blood lipids (total cholesterol, LDL, HDL, ratio of total cholesterol to HDL), biomarkers of renal and liver organ function (albumin and creatinine) and wide-range C-reactive protein (CRP) as a non-specific biomarker for inflammation. These biomarkers represent widely used indicators of health that are individually or cumulatively recognized as risk factors for age-related diseases among prime-aged and elderly individuals. Quantile regressions are used to estimate the age-gradient and the within-day variation of each biomarker distribution. Differences in biomarker levels by socioeconomic status are investigated using descriptive and multivariate statistics.

**Results:**

Overall, the number of significant associations between the biomarkers and socioeconomic measures is very modest. None of the biomarkers significantly varies with schooling. Except for CRP where being married is weakly associated with lower risk of having an elevated CRP level, marriage is not associated with the biomarkers measured in the MLSFH. Similarly, being Muslim is associated with a lower risk of having elevated CRP but otherwise religion does not predict being in the high-risk quartiles of any of the MLSFH biomarkers. Wealth does not predict being in the high-risk quartile of any of the MLSFH biomarkers, with the exception of a weak effect on creatinine. Being overweight or obese is associated with increased likelihood of being in the high-risk quartile for cholesterol, Chol/HDL ratio, and LDL.

**Conclusions:**

The results provide only weak evidence for variation of the biomarkers by socioeconomic indicators in a poor Malawian context. Our findings underscore the need for further research to understand the determinants of health outcomes in a poor low-income context such as rural Malawi.

## Background

Biomarker-based health indicators of physiological functioning represent a critical link for understanding the relationship between socioeconomic status (SES) and disease presentation because they can reveal common biological pathways between health and its socioeconomic and environmental determinants [1,2]. For instance, some studies have argued that individuals of low socioeconomic status have a higher prevalence of sub-clinical markers of disease risk [3], and longitudinal studies of cardiovascular disease (CVD) reveal that the individual’s relative rank on the biomarkers for CVD such as lipids or blood pressure tends to remain stable throughout the life course [4-6]. This suggests that even if currently measured biomarkers do not reveal a present clinical case, they may nevertheless represent a useful tool to identify individuals who are high risk for developing a disease [4]. Currently, however, the evidence about the relationship between SES and biomarkers of physiological health is mixed and derived primarily from studies in developed contexts. For instance, Rosero-Bixby et al. [7] found that metabolic conditions such as diabetes and cholesterol are not associated with SES, while hypertension and obesity worsen with higher SES. In a comparative multi-country study, Goldman et al. [8] observed generally negative and significant associations between education and different biomarkers in the United States (U.S.), but non-systematic and only weak associations in Taiwan and Costa Rica. Alley et al. [9] showed variation of C-reactive protein (CRP) by SES only at very high levels above 10 mg/l, but no difference at moderate or high levels of CRP, suggesting a non-linear relationship between inflammation and SES, at least in the U.S. context. Among the few studies in African contexts, Rossi et al. [10] found that serum concentration of high-sensitivity CRP was significantly associated with sex, several cardiovascular risk factors, and selected renal function markers in a Seychelles population. Studies have also suggested that the association between SES and biomarkers of health is stronger in developed than in the less-developed contexts, possibly due to the higher levels of social stratification in the industrialized world [8]. In addition, differences in nutritional patterns, ethnic origin, or exposure to environmental pathogens can potentially alter hematological and immunologic indicators and thus contribute to the variation of biomarkers between African and Western populations [11].

The present study contributes to the emerging literature on biomarker-based health indicators in sub-Saharan Africa (SSA) by analyzing SES differentials in several biomarkers collected as part of the Malawi Longitudinal Study of Families and Health (MLSFH) in the southern region of Malawi (Balaka). The objective of this analysis is to document the relationship between these biomarker-based indicators of health and socioeconomic status (SES) in a low-income African population where the cumulative effects of exposure to multiple stressors on physiological functions and health in general are expected to be highly detrimental for the well-being of individuals.

## Methods

### Study context

The MLSFH is a longitudinal study of the rural population in Malawi that provides an exceptional record of the social, economic, and health conditions in one of the world’s poorest nations. The MLSFH is based in three districts in rural Malawi that have been the study sites since 1998: Rumphi in the north, Mchinji in the center, and Balaka in the south. Respondents ( *N*_2008_≈4,000) are evenly split among the three study locations and clustered in 121 villages. The study population is broadly representative of the overall rural population in Malawi [12], and is similar in many socioeconomic and health conditions to other low-income countries in SSA [13]. MLSFH rounds were collected in 1998, 2001, 2004, 2006, 2008, and 2010. All three MLSFH sites are rural, and subsistence agriculture is the predominant economic activity among study participants. Balaka, which is the site for which biomarkers were collected in 2009, follows a matrilineal system of kinship and lineage system, and it is primarily inhabited by Lomwes and Yaos and has thus the highest proportion of Muslims among MLSFH regions. The Balaka region also exhibits a lower age of sexual debut and larger numbers of lifetime sexual partners than the other MLSFH study regions, and residents tend to be less educated and poorer than those living in the north, leading to higher levels of migration. HIV/AIDS prevalence in the southern region is significantly higher than in the northern and central region [14].

### MLSFH biomarkers and SES indicators

Our analyses focus on three groups of biomarkers: blood lipids (total cholesterol, high-density lipoprotein [HDL], low-density lipoprotein [LDL], and the ratio of total cholesterol to HDL), biomarkers of renal and liver organ function (albumin and creatinine), and wide-range C-reactive protein (CRP) as commonly used and reliable indicator of non-specific inflammation. The three groups represent a fairly broad set of biomarkers that embody multiple physiological processes and have individually as well as cumulatively been linked to important age-related health outcomes, including cardiovascular diseases, cognitive decline, physical performance, and death [15-17]. The selected biomarkers also represent commonly used biological indicators with demonstrated analytical power in population-based studies from both developed and less-developed countries [11,18-27]. For example, lipids are widely considered a risk factor for cardiovascular disease in the developed and developing countries [26-31], and the ratio of total cholesterol to HDL is a predictor of ischemic heart disease risk in asymptomatic individuals [23,32,33]. Low concentrations of albumin have been positively related to coronary artery disease and are also correlated with inflammation and malnutrition, while high levels are positively correlated with dehydration. In HIV-positive or malnourished individuals—both conditions are frequently occurring and co-existing in the Malawian rural population and SSA in general—creatinine levels may be elevated. In addition, renal diseases, and specifically chronic kidney disease, are among the leading causes of morbidity and mortality worldwide, and are understudied in SSA contexts [34-37].

SES indicators available in the MLSFH include: (*i*) respondent’s level of formal education (measured as no schooling, primary, and secondary schooling); and (*ii*) wealth indicators such as having a house covered with a metal roof and the wealth tertile based on an index constructed from dwelling characteristics and ownership of household durable assets using principal component analyses [38]. In addition, our analyses include other relevant aspects of the respondent’s demographic and socioeconomic context such as the respondent’s marital status (coded as married versus non-married) and religious affiliation (Christian, Muslim, and others). We also include body mass index (BMI) in the analysis since it is considered as a reliable indicator of current health problems (e.g., malnutrition, presence of HIV infection, and others).

### Data collection

The MLSFH collected blood-serum biomarker data along with a short survey for about 980 randomly selected respondents in the southern region of Balaka in January–February 2009. The details of the MLSFH biomarker collection are described in a companion paper [39], and IRB approval was obtained from the University of Pennsylvania and the Malawi National Health Sciences Research Council (NHSRC). As stipulated in these IRB protocols and the general regulations for conducting human subjects research, informed consent was obtained from all study participants prior to the participation in this study. The consent form clearly stated that the data are collected as part of a research project. The Balaka region was chosen for the MLSFH biomarker collection because of its relatively high HIV prevalence. All participants for the MLSFH biomarker sample were selected from MLSFH respondents who were successfully interviewed during the 2008 MLSFH wave, and the 2009 MLSFH biomarker sample is linked to all prior and subsequent MLSFH data collected for this study population. The target sample for the MLSFH biomarker collection was selected in two stages from the MLSFH respondent database. First, all respondents who were found HIV positive in a previous MLSFH round were included in the sample. Second, a random sample of approximately 1,500 respondents (aged ≥ 18 years) was drawn from the 2,500 MLSFH respondents residing in Balaka. The biomarker and survey data collection was conducted at respondents’ homes. Because of weather obstacles (rainy season), high levels of work-related migration in this region, and other temporary absences that resulted in failures to re-contact MLSFH respondents, we were able to successfully contact 1,031 individuals in the target sample. These individuals were offered to participate in the MLSFH biomarker study. Forty-nine respondents (4.7%) refused to participate, and the MLSFH successfully collected biomarker specimens for 982 respondents (95.2% of contacted individuals). Sixty-two study participants had previously tested positive for HIV (7.3% among those with known HIV status).

The MLSFH biomarker data collection used LabAnywhere kits (LabAnywhere, Harlem, the Netherlands; formerly known as Demecal) that require only a few drops of blood harvested from a lancet puncture of a sanitized fingertip. The reliability, sensitivity, and specificity of the test kits have been demonstrated by LabAnywhere in the Netherlands, and the applications of test-specific recovery factors yielded a good correlation with results of venous blood samples [40]. The LabAnywhere technology used in our study offers several advantages over the other common means of collecting blood samples in population-based studies such as dried blood spots (DBS) or venipuncture [41], including the extraction of blood plasma in fieldwork contexts with minimal discomfort for participants and the ability to conduct up to 16 assays with each sample. To collect the specimen for the LabAnywhere kits at respondents’ homes, the MLSFH recruited a team of 25 individuals who had previously been trained by the Malawian Government in finger prick blood collection as part of HIV voluntary counseling and testing. These biomarker collectors underwent an additional one-week of training in the use of the LabAnywhere kits, and each collector then completed about two to four home-based data biomarker and survey collections per day during fieldwork. While in the field during the day, the collected specimen were stored in a cooler. Upon returning from the field each day, the biomarker coordinator checked all samples to verify that they were collected and labeled properly; all plasma samples were stored in a -20°C freezer until they were shipped to the LabAnywhere laboratory on a weekly schedule. Prior to the shipment, all biomarker samples were cross-checked with field records. Shipment was via DHL from Malawi to the LabAnywhere laboratory in the Netherlands. The samples were packed in a special cooler with ice packs provided by LabAnywhere, which were designed specifically for transporting the frozen blood samples, including minimum/maximum thermometers to monitor the cooling conditions. LabAnywhere was able to analyze 910 (92.7%) of the 982 samples they received. None were discarded because of inadequate temperature control. The duration between the collection of each specimen and the analysis by LabAnywhere was almost always less than two weeks.

### Analytical approach

Descriptive statistics are used to present the distribution of each biomarker by gender. Quantile regressions of the 25th, 50th (median), and 75th percentile of each biomarker distribution on age are used to estimate the age-gradient of each biomarker distribution. Analogous quantile regressions on the time (hour) of the data collection were used to investigate intra-day variation in all biomarker distribution. To investigate differences in biomarker levels by socioeconomic characteristics, we follow the analytical approach of Dowd and Goldman [42] and apply logistic regressions using as dependent variable whether the biomarker value falls into the highest quartile of the observed distribution. This approach is advantageous compared to a linear specification if a risk for disease is associated with very low or very high values of the biomarkers; this approach is also relatively robust with respect to outliers in the biomarker distributions. Depending on the biomarker of interest, the highest-risk quartile can correspond to either low or high values. For instance, for total cholesterol, HDL, creatinine, CRP, and the ratio of total cholesterol to HDL the highest quartile corresponds to the 75th percentile of the distribution, while for albumin it corresponds to the 25th percentile. The logistic regression analyses are pooled for men and women to increase the statistical power of our analysis. All models control for sex of the respondents, age group (separate for males/females), and currently pregnant (females only). The results obtained from these logistic regression analyses with respect to biomarker differences by socioeconomic characteristics are identical with those obtained from multivariate linear regression using the log biomarker values as outcome (Additional file 1).

## Results and discussion

Descriptive statistics of the study population in Balaka are shown in Table 1. The average age of females included in the analysis is about 42 years, while men are on average 1 year older. The majority of the respondents (80% of women and 90% of men) are currently married. The Balaka region is predominantly Muslim and this is reflected in our sample: about 70% of the respondents are Muslim, while the remaining 30% are either Christian, belong to other religions, or are without religious affiliation. Women are on average less educated than men. For instance, 58% of women do not have any formal education, but only 32% of men fall into this category. Only 3% of women, but twice as many men, have secondary level of schooling. The majority of the respondents (80% of women and 84% of men) have normal body mass index as measured in 2008, the year prior the biomarker data collection. The data show that overweight and obesity are not common at all in rural Malawi, and only 7% of all respondents are overweight and 1% are obese, with both more prevalent among women than men. About 60% of the respondents rate their health status as being either very good or excellent, and only 16% of women and 10% of men rate it as being fair or poor. The majority of the MLSFH respondents reports better or much better health relative to others from the same sex and age group in the village.

**Table 1:** Summary statistics for the study population

	**Females**	**Males**	**Total**
	**mean**	**mean**	**mean**
	**(sd)**	**(sd)**	**(sd)**
# of observations	571	335	906
Age (in 2008)	42.17	43.54	42.68
	(17.75)	(16.87)	(17.43)
Married (in 2008)	0.76	0.89	0.81
Muslim	0.69	0.71	0.70
2008 level of education			
*No school*	0.57	0.32	0.48
*Primary level*	0.40	0.62	0.48
*Secondary level*	0.03	0.06	0.04
Body mass index (BMI) (2008)			
*Underweight* ( BMI<f.5)	0.14	0.12	0.14
*Normal* (18.5≤BMI<25)	0.75	0.83	0.78
*Overweight* ( 25≤BMI<f)	0.09	0.04	0.07
*Obese* ( BMI≥30)	0.02	0.01	0.01
Subjective health			
*Fair/Poor*	0.16	0.10	0.14
*Good*	0.31	0.19	0.26
*Very good*	0.28	0.30	0.29
*Excellent*	0.26	0.41	0.31
Relative health to others in village			
*Worse*	0.07	0.05	0.06
*Same*	0.31	0.28	0.30
*Better*	0.50	0.41	0.47
*Much better*	0.12	0.26	0.17
Number of recent econ shocks	2.00	1.88	1.95
	(0.91)	(0.95)	(0.92)

Summary statistics of the distribution of the biomarkers of interest are shown in Table 2. The distribution of the biomarkers deviates substantially from the distributions observed in the U.S. and other Western populations, and the observed values in our sample fall almost without exception below the clinically established levels used in industrialized countries to identify individuals at risk for adverse health outcomes [39]. This pattern is not entirely unexpected since, for instance, changes in lipoproteins are noted to occur during an acute-phase reaction to inflammation that is common in Malawi [43]. Similarly, inflammation and acute phase proteins may alter/reverse cholesterol transport by HDL [44]. As a consequence of the distribution of the biomarker levels in Table 2, a very low number of respondents can be characterized as high risk based on established clinical cutpoints for these biomarkers. In a prior study describing the methodology of the data collection, we tested the validity of our measurement approach and showed that this distribution of the biomarkers is not an artifact of measurement issues or problems [39], but it is similar to patterns observed in other low-income populations such as the Tsimane in Bolivia [45-47] or the Yakuts in Siberia who are, for instance, also characterized by very low CRP levels compared to the U.S. population samples [48,49].

**Table 2:** Summary statistics for the biomarker-based health indicators

	**N**	**Mean**	**std.**	**Percentiles**			**Age-Gradient**
				**25th**	**50th**	**75th**	**of median**
***Total cholesterol (Chol) (mg/dL)***							
Female	571	115.1	29.8	92.7	112.0	135.1	0.43 ^∗∗^
Male	336	103.5	37.9	83.0	100.4	119.7	0.64 ^∗∗^
Total	907	110.8	33.5	88.8	108.1	131.3	0.36 ^∗∗^
***High-density cholesterol (HDL) (mg/dL)***							
Female	571	33.4	11.6	27.0	30.9	42.5	0.00
Male	336	28.4	9.89	23.2	27.0	34.7	0.18 ^∗∗^
Total	907	31.6	11.2	23.2	30.9	38.6	-0.06
***Chol/HDL Ratio***							
Female	571	3.78	1.57	2.87	3.45	4.22	0.014 ^∗∗^
Male	336	3.97	1.73	3.00	3.63	4.40	0.002
Total	907	3.85	1.63	2.90	3.50	4.29	0.005 ^∗^
***Low-density cholesterol (LDL) (mg/dL)***							
Female	571	61.9	23.1	46.3	57.9	77.2	0.30 ^∗∗^
Male	336	53.9	28.5	38.6	50.2	65.6	0.45 ^∗∗^
Total	907	58.9	25.5	42.5	57.9	73.4	0.20 ^∗^
***Creatinine (mg/dL)***							
Female	571	0.67	0.17	0.54	0.66	0.76	0.002 ^∗∗^
Male	336	0.84	0.19	0.71	0.81	0.94	0.000
Total	907	0.73	0.20	0.60	0.71	0.84	0.002 ^∗^
***Albumin (g/dL)***							
Female	571	3.66	0.49	3.39	3.66	3.94	-0.003 ^∗∗^
Male	336	3.55	0.51	3.25	3.52	3.84	-0.008 ^∗∗^
Total	907	3.62	0.50	3.33	3.62	3.91	-0.006 ^+^
***C-reactive protein (CRP)***							
Female	561	3.82	11.0	0.20	0.50	2.30	0.006 ^∗^
Male	332	4.99	12.2	0.20	0.80	3.00	0.013 ^∗^
Total	893	4.26	11.5	0.20	0.60	2.60	0.002 ^∗∗^

Age gradient of median is the coef of a quantile regression of the 50th percentile on age. *p*-values: + ***p<0.10***, ^***∗***^***p<0.05***, ^***∗∗***^***p<0.01***.

**Figure 1:**
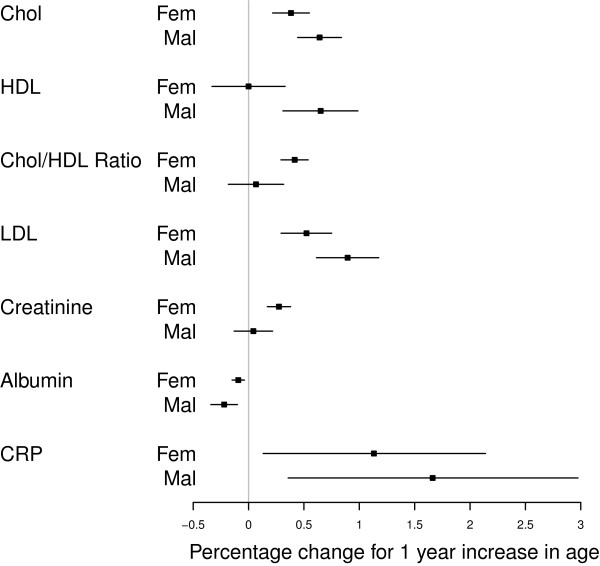
Relative change in median value of biomarker for a 1-year increase in age (separate analyses for females and males).

The analysis of the age-gradient reveals that the distribution of cholesterol is shifted upward for older individuals (Table 2 and Figure 1); the same is the case for HDL (men only), Chol/HDL Ratio (women only), LDL, and creatinine (women only). Albumin declines with age. Similar age-gradients were also found at the 25th and 75th percentile of the biomarker distributions (results not reported in detail). Even where the age-gradient is statistically significant, age explains only a very small fraction of the variation in the biomarkers: Pseudo-R^2^ of all age-gradient regressions are below .05 in all cases except for some regressions for cholesterol and one for LDL where the Pseudo-R^2^ reaches values between .07–.09. Quantile regressions of the 25th, 50th, and 75th percentile on dummies for the hour of biomarker collection during each day and gender (not reported in detail) show that, with the exception of the 25th percentile for albumin, there is no systematic time variation of the biomarker distributions within each fieldwork day: the null hypotheses that 25th, 50th, and 75th percentile does not vary by hour of day is never rejected at the 5 percent level, with exception of the 25th percentile for albumin where a weak inverse U-shape is detected. HDL and albumin levels are significantly lower among HIV+ as compared to HIV– respondents ( *p*<.01), while CRP levels and the Chol/HDL ratio are elevated among HIV+ as compared to HIV– respondents ( *p*<.01).

Table 3 shows odds ratios for the probability that a respondent is in the highest-risk quartiles of the biomarker distribution by schooling, marital status, religion, wealth, and BMI. Overall, the number of significant associations between the biomarkers and socioeconomic measures is very modest. None of the biomarkers significantly varies with schooling, despite the fact that schooling has been shown to be an important health and socioeconomic indicator in this and related contexts [50]. Despite the widely documented association between marriage and better health [51], marriage is not associated with the biomarkers measured in the MLSFH; the only exception is CRP where being married is weakly associated with lower risk of having an elevated CRP level (at 10% statistical significance). The same lack of a strong association is found for religion, where being Muslim is associated with a lower risk of having elevated CRP but otherwise does not predict being in the high-risk quartiles of any of the MLSFH biomarkers. Despite widely documented associations between wealth and health [38,52], wealth does not predict being in the high-risk quartile of any of the MLSFH biomarkers (only one association between creatinine and the highest wealth tertile is significant at the 10% level). The lack of strong associations between the biomarker levels and socioeconomic indicators such as schooling, marital status, religion, and wealth is also found in identical fashion in multivariate regression analyses using the log biomarker levels as continuous outcomes (Additional file 1: Table S1). Consistent with research that increasingly emphasizes elevated levels of BMI as a health concern also in developing countries [53,54], being overweight or obese—which is the case for 8.7% of the MLSFH respondents in the analyses sample—is associated with increased likelihood of being in the high-risk quartile for cholesterol, Chol/HDL ratio, LDL (Table 3). For all biomarkers, the point estimates suggest that being underweight is associated with a reduced likelihood, and being overweight is associated with an increased likelihood of being in the high-risk quartile of the biomarker distribution, but except where noted above, the estimates are not significant at the 5% level.

**Table 3:** Odds ratios for the probability of being in the highest risk quartiles of the biomarker distributions: BMI, subjective health and other self-reported health measures (logistic regression models, both sexes combined)

	**Cholesterol**	**HDL**	**Chol/HDL ratio**	**LDL**	**Creatinine**	**CRP**	**Albumin**
**Model 1: Schooling**							
No Schooling	ref.	ref.	ref.	ref.	ref.	ref.	ref.
Primary Schooling	1.04	1.08	1.33	1.13	1.14	1.27	0.91
	[0.70,1.55]	[0.75,1.55]	[0.91,1.95]	[0.76,1.66]	[0.75,1.72]	[0.87,1.85]	[0.63,1.31]
Secondary Schooling	0.79	0.62	1.39	0.54	1.98	1.22	0.62
	[0.25,2.56]	[0.25,1.50]	[0.56,3.45]	[0.15,1.94]	[0.79,4.97]	[0.50,2.97]	[0.22,1.76]
**Model 2: Marital status**							
Married (in 2008)	1.11	0.80	0.94	1.08	0.99	0.65 ^+^	1.02
	[0.69,1.79]	[0.52,1.22]	[0.61,1.46]	[0.68,1.72]	[0.60,1.63]	[0.42,1.01]	[0.64,1.61]
**Model 3: Religion (major groups)**							
Christian/Other/None	ref.	ref.	ref.	ref.	ref.	ref.	ref.
Muslim	1.18	0.89	0.95	1.07	0.68 ^+^	0.68 ^∗^	0.85
	[0.80,1.73]	[0.63,1.24]	[0.67,1.36]	[0.74,1.55]	[0.47,1.00]	[0.48,0.95]	[0.60,1.20]
**Model 4: Wealth (based on asset-based wealth tertiles)**							
1st (poorest)	1.05	1.17	0.79	1.09	1.38	0.77	0.97
	[0.68,1.62]	[0.79,1.72]	[0.52,1.19]	[0.71,1.67]	[0.88,2.15]	[0.52,1.16]	[0.66,1.44]
2nd (middle)	ref.	ref.	ref.	ref.	ref.	ref.	ref.
3th (wealthiest)	1.08	1.25	1.15	1.30	1.49 ^+^	1.02	0.75
	[0.70,1.67]	[0.85,1.85]	[0.77,1.71]	[0.86,1.99]	[0.94,2.34]	[0.68,1.52]	[0.50,1.13]
**Model 5: Body mass index (BMI) (reference category: normal)**							
Underweight	0.81	0.74	0.63	0.77	0.55 ^+^	0.69	1.42
	[0.45,1.46]	[0.42,1.31]	[0.34,1.16]	[0.43,1.39]	[0.28,1.06]	[0.39,1.24]	[0.85,2.36]
Normal	ref.	ref.	ref.	ref.	ref.	ref.	ref.
Overwght/obese	2.11 ^∗^	1.14	2.65 ^∗∗^	2.65 ^∗∗^	1.39	1.35	0.71
	[1.14,3.90]	[0.59,2.18]	[1.46,4.81]	[1.46,4.81]	[0.68,2.86]	[0.69,2.62]	[0.34,1.47]
BMI missing	1.00	1.54 ^∗^	1.52 ^∗^	1.20	0.79	1.13	1.01
	[0.66,1.51]	[1.09,2.16]	[1.06,2.18]	[0.80,1.79]	[0.53,1.17]	[0.78,1.62]	[0.70,1.46]

*p*-values: + ***p<0.10***, ^***∗***^***p<0.05***, ^***∗∗***^***p<0.01***. 95% Confidence intervals in parentheses. Models control for gender, age group (separate for males/females), and currently pregnant (females only).

The lack of significant associations between the MLSFH biomarkers and SES is not due to a lack of statistical power. Our analyses document significant age patterns for several biomarkers, significant associations between BMI and several biomarkers (cholesterol, Chol/HDL ratio, LDL levels), significant differences in the expected direction in four out of the seven biomarkers between HIV+ and HIV– respondents, and associations between CRP and marital status and religion that are in the expected directions. These statistically significant findings suggest that our analyses are in principle adequately powered to detect differences in the MLSFH biomarkers by SES and other indicators (which is consistent with the power calculations used in designing this study). The failure to document important socioeconomic variation in many of our analyses therefore seems not due to an inadequate sample size, but rather due to a lack of strong associations between the MLSFH biomarkers and our socioeconomic indicators.

## Conclusions

Biomarker-based health indicators of physiological functioning represent a critical link for understanding the relationship between socioeconomic status (SES) and disease presentation. And yet, current evidence about SES differentials in biomarker-based health measures among prime-aged and elderly individuals remains inconclusive. Our analyses focus on commonly used biomarkers of cardiovascular risk, non-specific inflammation, and renal/liver function that are individually or cumulatively recognized as risk factors for age-related diseases among prime-aged and elderly individuals: total cholesterol, LDL, HDL, ratio of total cholesterol to HDL, albumin, creatinine, and wide-range CRP. And while a large body of research has documented differences in these biomarkers by socioeconomic indicators in other contexts [7-11,42,55-60], the variation of these biomarkers in the Malawian—or similar poor high-morbidity contexts in SSA—is still not well established. However, analyses of the SES variation in these biomarker-based health indicators are an important addition to the existing literature on socioeconomic health differentials in adult populations because most existing studies in SSA are often based on self-reported measures of health and disability, and only for HIV and some other infectious diseases, on biomarkers. At this point, little is known from biomarker-based studies about the levels, variations, and determinants of cardiovascular risk, non-specific inflammation, and renal/liver function in poor high-morbidity SSA populations. And as the epidemiological transition in SSA progresses and chronic diseases constitute a growing proportion of the SSA disease burden [61], these questions will become increasingly important. And quite possibly, the story emerging from biomarker-based indicators of physiological functions and health could point to a more complex interpretation of the determinants of adults health in contexts such as Malawi than conclusions that are often based on self-reported health measures and/or are frequently focused on infectious diseases.

Our analyses are also important from a methodological standpoint. For instance, to which extent are biomarkers such as those collected as part of the MLSFH suitable for identifying health differentials in poor high-morbidity SSA contexts such as Malawi? And to what extent do they provide comparable data on physiological functioning? And do these biomarkers exhibit the same socioeconomic gradients in Malawi that have been documented for these biomarkers in other contexts, or do they exhibit the same gradients as other (mostly self-reported) health indicators?

The key findings of our study point to an important “puzzle”: Despite strong hypothesis for the existence of SES differentials in health (and the MLSFH biomarkers in particular), our results provide only weak evidence for variation in the MLSFH biomarkers for cardiovascular risk, non-specific inflammation, and renal or liver functioning by socioeconomic status. Moreover, only for a small proportions of respondents do any of the MLSFH biomarkers fall outside the limits of the normal clinical ranges as defined by Western standards [39]. Combined these findings are important because it has been suggested that biomarker-based indicators of health can provide a useful tool to model and understand the pathways of how socioeconomic characteristics relate and influence physiological functioning [62-64]. However, the lack of important SES variation and the shifted distribution of the MLSFH biomarker (Table 2) as compared to those observed in more developed contexts raises the possibility that specific contexts of individuals in poor high-morbidity SSA environments importantly affect the distribution of these biomarkers and their association with SES and other behavioral/contextual covariates. Our findings thus underscore the need for further research to understand the determinants of the levels and variations in biomarker-based health indicators that measure cardiovascular risk, inflammation and renal or liver function. In poor high-morbidity SSA contexts, the variation in these indicators is currently understudied. However, a better understanding of the determinants and variation of these biomarker-based health indicators can importantly contribute to an improved understanding of adult health that is construed more broadly than is currently the case in many studies that focus on either self-reported health measures or infectious diseases. Only if the usefulness and validity of such biomarkers can be established does it make sense to further expand biomarker-based health measures in population-based surveys in poor high-morbidity SSA contexts to obtain information about otherwise unobserved dimensions of health. And while the technological challenges for expanding biomarker-based health measures as part of population-based studies in SSA are increasingly surmountable, our findings highlight the potential ongoing challenges in interpreting and comparing these measures within SSA populations and across diverse cross-country contexts.

The strengths of this study include the population-based design; the availability of multiple biomarkers providing indicators of cardiovascular risk, non-specific inflammation, and renal/liver function; a home-based data collection; a high participation rate; and the integration in a longitudinal cohort study with extensive socioeconomic, demographic, and health information. Among the potential limitations of the present analyses is the fact that these results pertain to a small limited population from a discrete region in rural Malawi. Specifically, the analyses are based on data from the southern region of Balaka, an area that is predominantly Muslim. The sample size comprises about 900 individuals and is at most representative for this part of the country, but not of the entire Malawian population. It is possible that while the link between socioeconomic status and blood lipids, creatinine, albumin and wide-range CRP in this setting is similar to the observations in other developed countries, the pathways are different in this cultural setting and are not captured by these analyses. Moreover, while we measure biomarkers that are seen as a standard for health assessment in primarily Western elderly populations, they may not be the best measure for assessing health status in the SSA context. It is also possible that the associations between the biomarkers and the socioeconomic indicators may be confounded by other risk factors such as nutritional deficits and episodic malnutrition, high prevalence of smoking, and presence of co-infections such as HIV, tuberculosis, or endemic parasites. In the present study, we are however not able to control for these potentially confounding factors.

## Competing interests

The authors declare that they have no competing interests.

## Authors’ contributions

IVK took the lead in conducting the analyses and writing up the manuscript; she also contributed to the design of the MLSFH biomarker data collection in 2009. BS contributed to the design of the MLSFH biomarker collection and contributed to the analyses and writing up of the results. PA was the fieldwork director for the collection of the MLSFH biomarkers in 2009, and he contributed to the analyses and writing up of the results. Ben Chilima contributed to the design and the implementation of the 2009 MLSFH biomarker collection. HPK is the PI of the MLSFH; he contributed to the design of the MLSFH biomarker collection, and the analyses and writing up of the results for this paper. All authors read and approved the final manuscript.

## Supplementary Material

Additional file 1 Multivariate regression of log biomarker levels on selected socioeconomic indicators (both sexes combined). Click here for file
