# Supplementary material for: Association of blood lipids, creatinine, albumin, and CRP with socioeconomic status in Malawi
Source: Popul Health Metr. 2013 Feb 28;11:4. doi: 10.1186/1478-7954-11-4 (PMC3701600; doi:10.1186/1478-7954-11-4)
Supplement: Additional file 1 — Multivariate regression of log biomarker levels on selected socioeconomic indicators (both sexes combined). [file 1478-7954-11-4-S1.pdf]

Table A.1: Multivariate regression of log biomarker levels on selected socioeconomic indicators (both sexes combined)

	**Cholesterol**	**HDL**	**Chol/HDL** Ratio	**LDL**	**Creatinine**	**CRP**	**Albumin**
**Model 1: Schooling**							
No Schooling	ref.	ref.	ref.	ref.	ref.	ref.	ref.
Primary Schooling	0.035^{+}	0.029	0.0060	0.052	0.0025	0.18	0.0085
	[-0.0064,0.077]	[-0.033,0.092]	[-0.044,0.056]	[-0.014,0.12]	[-0.033,0.039]	[-0.12,0.48]	[-0.013,0.030]
Secondary Schooling	0.079	0.061	0.017	0.069	0.068	-0.013	0.017
	[-0.022,0.18]	[-0.090,0.21]	[-0.10,0.14]	[-0.090,0.23]	[-0.019,0.16]	[-0.75,0.72]	[-0.035,0.069]
**Model 2: Marital status**							
Married (in 2008)	0.025	0.038	-0.013	0.026	0.0056	-0.28	0.0054
	[-0.025,0.075]	[-0.037,0.11]	[-0.073,0.047]	[-0.052,0.10]	[-0.037,0.048]	[-0.64,0.084]	[-0.020,0.031]
**Model 3: Religion (major groups)**							
Christian/Other/None	ref.	ref.	ref.	ref.	ref.	ref.	ref.
Muslim	0.028	0.015	0.013	0.027	-0.015	-0.21	0.018^{+}
	[-0.011,0.067]	[-0.045,0.075]	[-0.034,0.061]	[-0.035,0.090]	[-0.049,0.019]	[-0.49,0.079]	[-0.0020,0.039]
**Model 4: Wealth (based on asset-based wealth tertiles)**							
1st (poorest)	0.0086	0.042	-0.033	0.0092	0.037^{+}	-0.093	-0.012
	[-0.036,0.053]	[-0.025,0.11]	[-0.086,0.020]	[-0.061,0.080]	[-0.0020,0.075]	[-0.42,0.23]	[-0.035,0.011]
2nd (middle)	ref.	ref.	ref.	ref.	ref.	ref.	ref.
3th (wealthiest)	0.013	-0.0058	0.019	0.035	0.027	-0.14	0.0091
	[-0.031,0.058]	[-0.073,0.061]	[-0.034,0.072]	[-0.036,0.11]	[-0.012,0.066]	[-0.46,0.19]	[-0.014,0.032]
**Model 5: Body Mass Index (BMI) (reference category: normal)**							
Underweight	-0.048	-0.00073	-0.047	-0.092^{+}	-0.075**	-0.056	-0.015
	[-0.11,0.012]	[-0.092,0.090]	[-0.12,0.025]	[-0.19,0.0027]	[-0.13,-0.023]	[-0.49,0.38]	[-0.046,0.017]
Normal	ref.	ref.	ref.	ref.	ref.	ref.	ref.
Overwght/Obese	0.077*	-0.056	0.13**	0.15**	0.056^{+}	0.71*	0.0093
	[0.0034,0.15]	[-0.17,0.054]	[0.046,0.22]	[0.038,0.27]	[-0.0076,0.12]	[0.16,1.26]	[-0.029,0.048]
BMI missing	-0.017	-0.057^{+}	0.040	-0.0071	-0.021	0.013	0.0020
	[-0.059,0.024]	[-0.12,0.0052]	[-0.0094,0.090]	[-0.073,0.058]	[-0.057,0.014]	[-0.29,0.31]	[-0.020,0.024]

Log values of the biomarkers are used as dependent variable to reduce skewness of the distributions; for 48 cases where the CRP variable equals zero because CRP was below detectable levels, log(.01) is used as the value of the dependent variable.
p-values: $^{+}$ $p < 0.10$, * $p < 0.05$, ** $p < 0.01$. 95% Confidence intervals in parentheses.
Models control for gender, age group (separate for males/females), and currently pregnant (females only).
